# Ilmenite-type Na_2_(Fe_2/3_Te_4/3_)O_6_

**DOI:** 10.1107/S2414314624004826

**Published:** 2024-05-31

**Authors:** Felix Eder, Matthias Weil

**Affiliations:** a TU Wien, Institute for Chemical Technologies and Analytics, Division of Structural Chemistry, Getreidemarkt 9/E164-05-1, 1060 Vienna, Austria; Goethe-Universität Frankfurt, Germany

**Keywords:** crystal structure, mixed occupancy, isotypism, close-packed structure

## Abstract

Na_2_(Fe_2/3_Te_4/3_)O_6_ (*Z* = 3) or Na_3_(FeTe_2_)O_9_ (*Z* = 2), tris­odium iron(III) ditellurium(VI) nona­oxide, adopts the ilmenite (FeTiO_3_, *Z* = 6) structure type with the Ti site (site symmetry 3.) replaced by Na and the Fe site (site symmetry 3.) replaced by a mixed-occupied (Fe^III^,Te^VI^) site in a Fe:Te ratio of 1:2. Whereas the [(Fe,Te)O_6_] octa­hedron is only slightly distorted, the [NaO_6_] octa­hedron shows much stronger distortions, as revealed by a larger spread of the bond lengths and some distortion parameters.

## Abstract 2

Na_2_(Fe_2/3_Te_4/3_)O_6_ was obtained under hydro­thermal conditions and adopts the ilmenite (FeTiO_3_) structure type.

## Graphical abstract

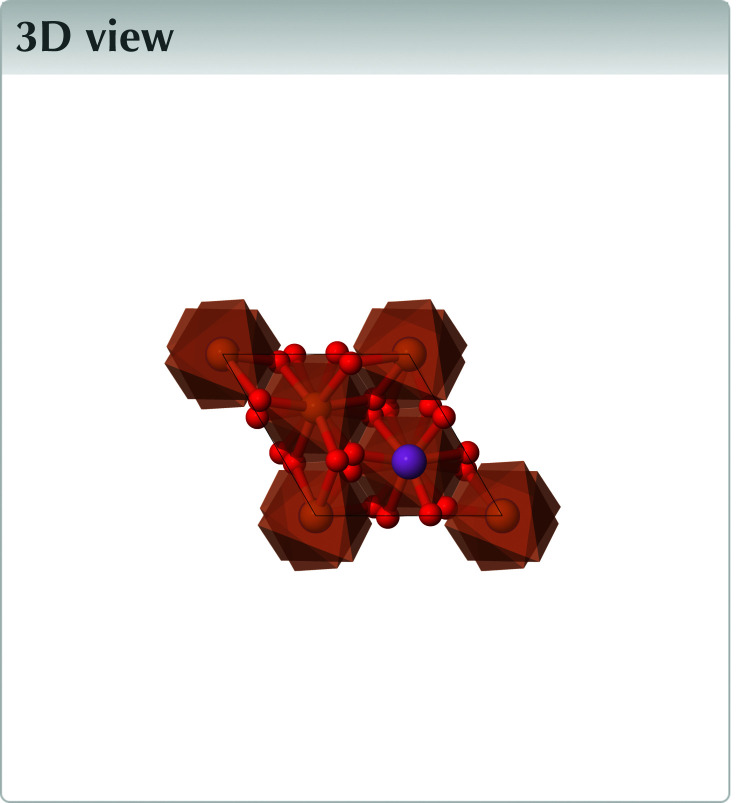

## Structure description

Crystals of Na_2_(Fe_2/3_Te_4/3_)O_6_ were inadvertently obtained during hydro­thermal synthesis attempts originally aiming at a phase with composition Na_12_Fe^III^_6_Te^VI^_4_O_27_·3H_2_O and for which possible relationships with the potassium phase K_12_Fe^III^_6_Te^VI^_4_O_27_·3H_2_O (Eder, 2023 ▸) were to be investigated.

Na_2_(Fe_2/3_Te_4/3_)O_6_ (*Z* = 3) or Na_3_(FeTe_2_)O_9_ (*Z* = 2) crystallizes in the ilmenite structure type (FeTiO_3_, *Z* = 6). The Na^+^ cations take the Ti sites and the occupationally disordered (Fe^III^/Te^VI^) atoms (ratio Fe^III^:Te^VI^ = 1:2) take the Fe sites of the ilmenite structure. The latter is a twofold superstructure of the corundum structure where two-thirds of the octa­hedral voids of the hexa­gonal close packed (hcp) structure defined by O atoms are occupied (Wells, 1975 ▸). The two types of metal sites in the ilmenite structure, both with site symmetry 3. (multiplicity 6, Wyckoff letter *c*), have a distorted octa­hedral oxygen environment. The [(Fe,Te)O_6_] octa­hedron is only slightly distorted, the [NaO_6_] octa­hedron more clearly as evidenced by their bond lengths distribution [(Fe,Te)—O = 1.951 (3) Å (3×), 1.993 (3) Å (3×); Na—O1 = 2.297 (3) Å (3×), 2.545 (4) Å (3×)], and by qu­anti­tative distortion parameters (Robinson *et al.*, 1971 ▸) [quadratic elongation: ([(Fe,Te)O_6_] = 1.018; [NaO_6_] = 1.062; angle variance: [(Fe,Te)O_6_] = 62.99°^2^; [NaO_6_] = 204.27°^2^]. The polyhedral volume of [(Fe,Te)O_6_] amounts to 9.950 Å^3^, and that of [NaO_6_] to 17.378 Å^3^ as calculated with the VOLCAL option in *PLATON* (Spek, 2020 ▸).

The only other Te-containing compounds adopting the ilmenite structure type deposited with the Inorganic Crystal Structure Database (ICSD, release 2023–1; Zagorac *et al.*, 2019 ▸) are Na_2_(Ti^IV^Te^VI^)O_6_ and *α*-Na_2_(Ge^IV^Te^VI^)O_6_ (Woodward *et al.*, 1999 ▸).

**Figure 1:**
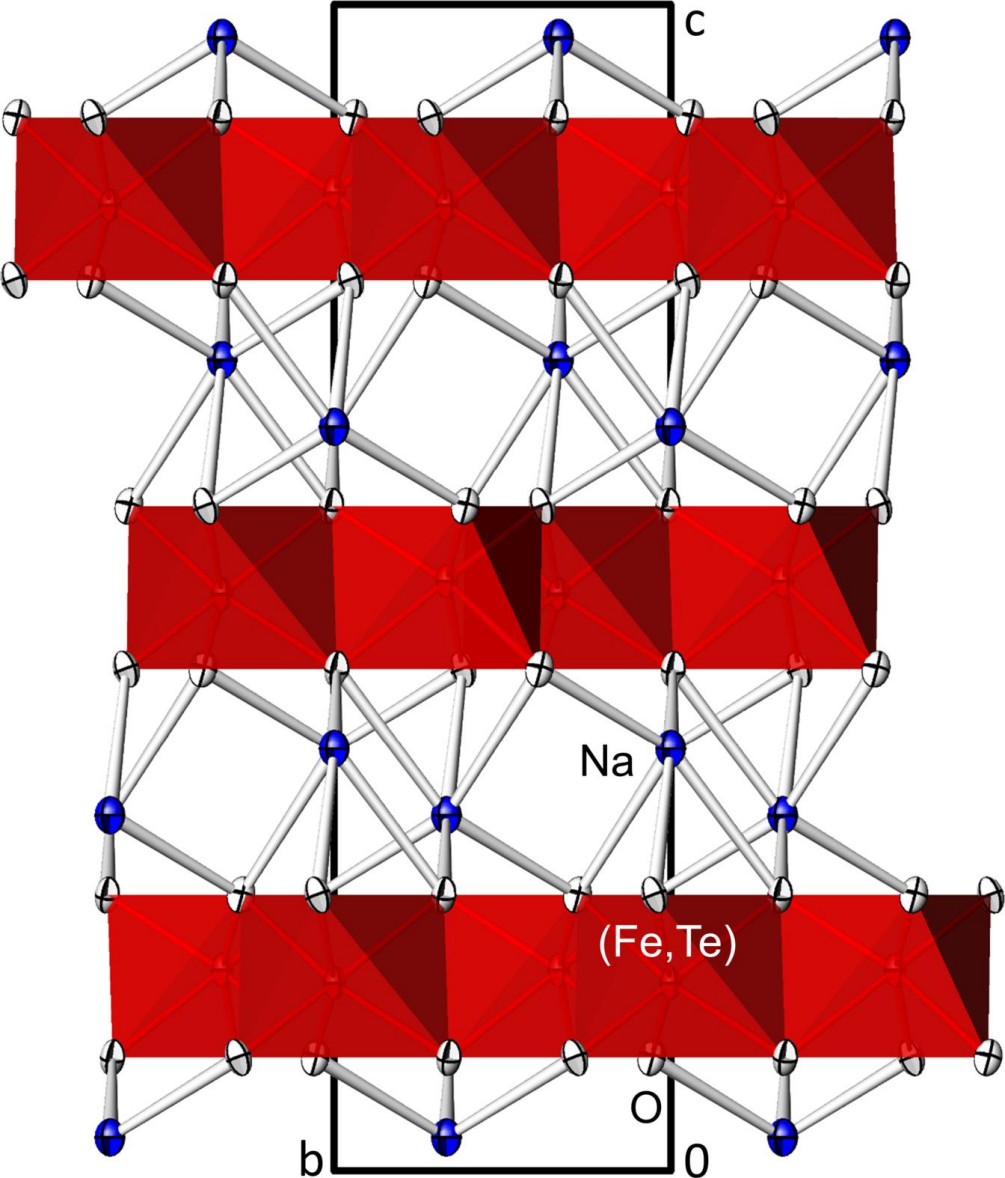
Crystal structure of ilmenite-type Na_2_(Fe_2/3_Te_4/3_)O_6_ in a projection along [ 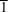 00] showing the layer stacking along [001]. [(Fe,Te)O_6_] units are shown in the polyhedral representation (red octa­hedra), Na atoms (blue) with bonds to the O atoms. Displacement ellipsoids are drawn at the 74% probability level.

The ilmenite-type crystal structure of Na_2_(Fe_2/3_Te_4/3_)O_6_ is shown in Figs. 1 ▸ and 2 ▸.

## Synthesis and crystallization

Hydro­thermal synthesis conditions were the same as detailed for garnet-type Na_3_Te_2_(FeO_4_)_3_ (Eder & Weil, 2023 ▸). Small amounts of yellowish (nearly colourless) crystals of Na_2_(Fe_2/3_Te_4/3_)O_6_ with a plate-like form were harvested from the reaction mixture that also contained very few colourless crystals of NaFe^III^(Te^IV^O_3_)_2_ (Weil & Stöger, 2008 ▸).

## Refinement

Crystal data, data collection and structure refinement details are summarized in Table 1 ▸.

**Figure 3:**
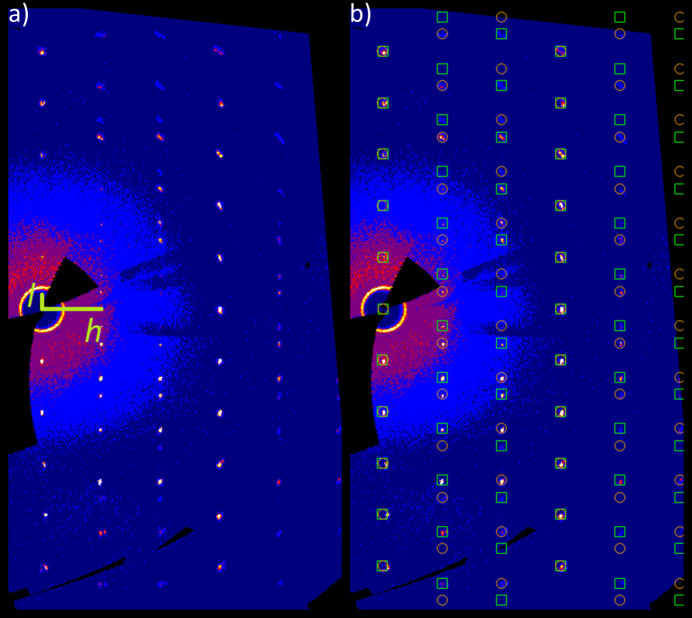
(*a*) Reconstructed reciprocal *h*0*l* plane of Na_2_(Fe_2/3_Te_4/3_)O_6_; (*b*) reflections belonging to the two twin domains are marked in green squares (domain 1) and orange circles (domain 2); reflections of the two domains overlap in every third row *h*.

All crystals under investigation were systematically twinned with at least two twin domains present. During the integrating and scaling process of the finally chosen crystal, a pseudo-Laue class of 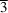 .*m* was suggested by *X-AREA* (Stoe, 2021 ▸) with similar *R*_int_ values compared to the Laue class corresponding to the actual structure ( 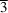 ). From this pseudo-symmetry imposed by the twinning, one possible twin law (*m*_(210)_) with a transformation of **a**, −**a**−**b**, **c** was derived. The intensity data were integrated on basis of a hexa­gonal primitive unit-cell of same dimensions to include the reflections of both twin domains (Fig. 3 ▸). By applying the twin law given above, the ratios of the respective domains refined to values of 0.540:0.460 (2). For the sake of charge-neutrality, the mixed-occupied (Fe^III^/Te^VI^) site was constrained to a ratio of 1:2 for Fe:Te. The two atom types located at this site were refined with common displacement parameters.

Structure data of Na_2_(Fe_2/3_Te_4/3_)O_6_ were standardized with *STRUCTURE-TIDY* (Gelato & Parthé, 1987 ▸).

## Supplementary Material

Crystal structure: contains datablock(s) I, general. DOI: 10.1107/S2414314624004826/bt4150sup1.cif

Structure factors: contains datablock(s) I. DOI: 10.1107/S2414314624004826/bt4150Isup2.hkl

CCDC reference: 2357540

Additional supporting information:  crystallographic information; 3D view; checkCIF report

## Figures and Tables

**Figure 2:**
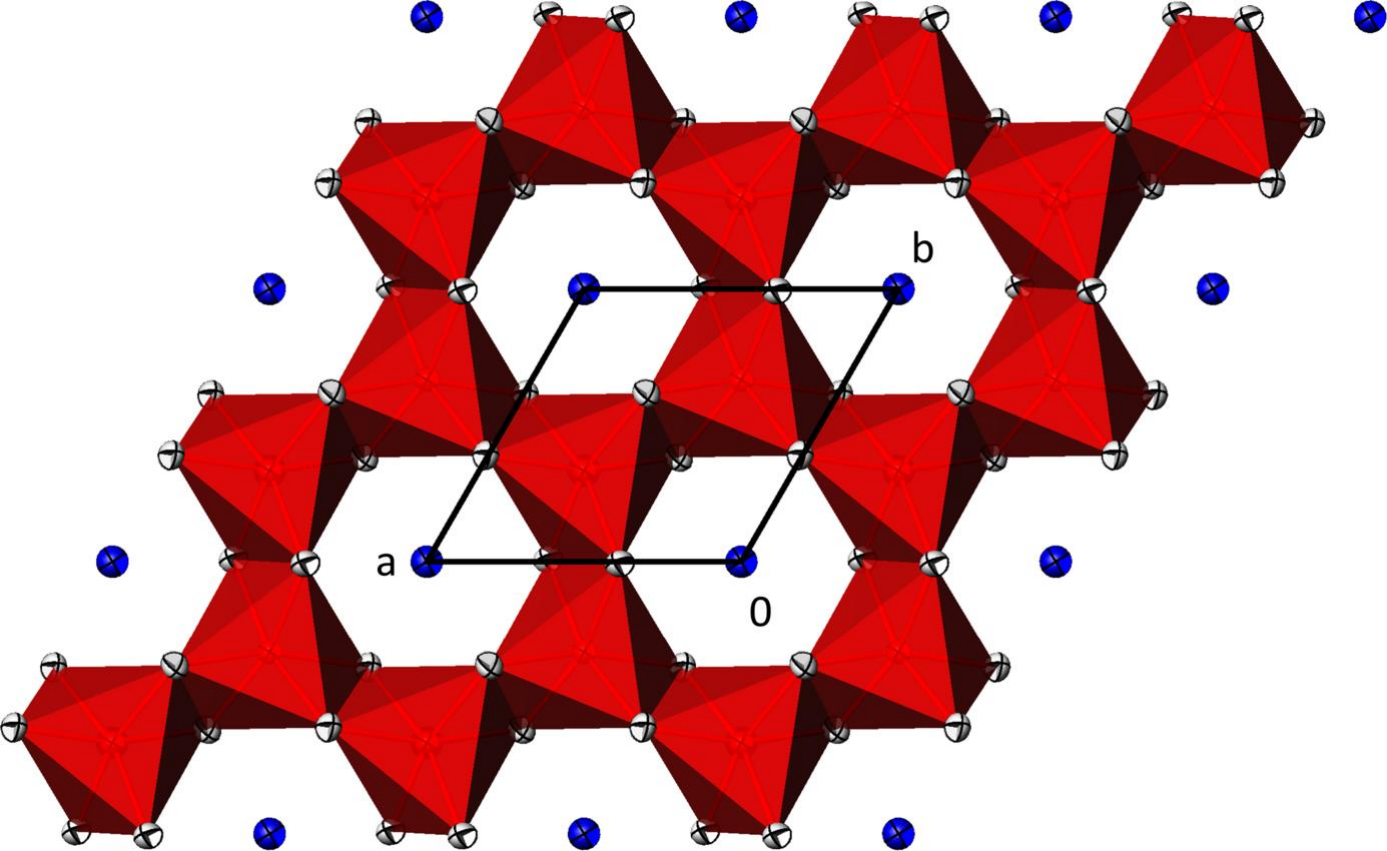
Projection of the crystal structure of Na_2_(Fe_2/3_Te_4/3_)O_6_ onto (001), showing only one layer of [(Fe,Te)O_6_] units (red polyhedra) and of Na atoms (blue). For clarity, Na—O bonds are omitted. Displacement ellipsoids are drawn at the 74% probability level.

**Table 1:** Experimental details

Crystal data	
Chemical formula	Na_2_(Fe_2/3_Te_4/3_)O_6_
*M*_r_	349.33
Crystal system, space group	Trigonal, *R* 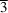 :*H*
Temperature (K)	300
*a*, *c* (Å)	5.2598 (8), 15.778 (3)
*V* (Å^3^)	378.02 (14)
*Z*	3
Radiation type	Mo *K*α
μ (mm^−1^)	9.76
Crystal size (mm)	0.05 × 0.04 × 0.01

Data collection	
Diffractometer	Stoe Stadivari
Absorption correction	Multi-scan (*LANA*; Koziskova *et al.*, 2016 ▸)
*T*_min_, *T*_max_	0.519, 0.596
No. of measured, independent and observed [*I* > 2σ(*I*)] reflections	3266, 454, 383
*R*_int_	0.043
(sin θ/λ)_max_ (Å^−1^)	0.731

Refinement	
*R*[*F* ^2^ > 2σ(*F* ^2^)], *wR*(*F* ^2^), *S*	0.027, 0.066, 1.03
No. of reflections	454
No. of parameters	17
Δρ_max_, Δρ_min_ (e Å^−3^)	1.64, −1.30

Computer programs: *X-AREA* (Stoe, 2021 ▸), *SHELXT* (Sheldrick, 2015*a* ▸), *SHELXL* Sheldrick, 2015*b* ▸), *ATOMS* (Dowty, 2006 ▸) and *publCIF* (Westrip, 2010 ▸).

## full crystallographic data

### Crystal data

**Table:**

Na_2_Fe_0.6666_Te_1.3333_O_6_	*D*_x_ = 4.604 Mg m^−^^3^
*M**_r_* = 349.33	Mo *K*α radiation, λ = 0.71073 Å
Trigonal, *R*3:*H*	Cell parameters from 3735 reflections
*a* = 5.2598 (8) Å	θ = 3.9–31.3°
*c* = 15.778 (3) Å	µ = 9.76 mm^−^^1^
*V* = 378.02 (14) Å^3^	*T* = 300 K
*Z* = 3	Plate, yellowish
*F*(000) = 470	0.05 × 0.04 × 0.01 mm

### Data collection

**Table:**

Stoe Stadivari diffractometer	383 reflections with *I* > 2σ(*I*)
Radiation source: Axo_Mo	*R*_int_ = 0.043
rotation method, ω scans	θ_max_ = 31.3°, θ_min_ = 4.7°
Absorption correction: multi-scan (*LANA*; Koziskova *et al.*, 2016)	*h* = −4→7
*T*_min_ = 0.519, *T*_max_ = 0.596	*k* = −7→5
3266 measured reflections	*l* = −22→23
454 independent reflections	

### Refinement

**Table:**

Refinement on *F*^2^	17 parameters
Least-squares matrix: full	0 restraints
*R*[*F*^2^ > 2σ(*F*^2^)] = 0.027	*w* = 1/[σ^2^(*F*_o_^2^) + (0.0446*P*)^2^] where *P* = (*F*_o_^2^ + 2*F*_c_^2^)/3
*wR*(*F*^2^) = 0.066	(Δ/σ)_max_ < 0.001
*S* = 1.03	Δρ_max_ = 1.64 e Å^−^^3^
454 reflections	Δρ_min_ = −1.30 e Å^−^^3^

### Special details

**Table:**

Geometry. All esds (except the esd in the dihedral angle between two l.s. planes) are estimated using the full covariance matrix. The cell esds are taken into account individually in the estimation of esds in distances, angles and torsion angles; correlations between esds in cell parameters are only used when they are defined by crystal symmetry. An approximate (isotropic) treatment of cell esds is used for estimating esds involving l.s. planes.
Refinement. Refined as a 2-component inversion twin.

### Fractional atomic coordinates and isotropic or equivalent isotropic displacement parameters (Å^2^)

**Table:**

	*x*	*y*	*z*	*U*_iso_*/*U*_eq_	Occ. (<1)
Te	0.000000	0.000000	0.16114 (4)	0.0138 (2)	0.6667
Fe	0.000000	0.000000	0.16114 (4)	0.0138 (2)	0.3333
Na	0.000000	0.000000	0.3619 (2)	0.0215 (6)	
O	0.3411 (6)	0.0563 (7)	0.0969 (2)	0.0181 (6)	

### Atomic displacement parameters (Å^2^)

**Table:**

	*U*^11^	*U*^22^	*U*^33^	*U*^12^	*U*^13^	*U*^23^
Te	0.0101 (2)	0.0101 (2)	0.0212 (3)	0.00503 (11)	0.000	0.000
Fe	0.0101 (2)	0.0101 (2)	0.0212 (3)	0.00503 (11)	0.000	0.000
Na	0.0183 (9)	0.0183 (9)	0.0278 (15)	0.0091 (4)	0.000	0.000
O	0.0126 (14)	0.0156 (13)	0.0260 (15)	0.0068 (11)	0.0020 (11)	−0.0026 (12)

### Geometric parameters (Å, º)

**Table:**

Te—O	1.951 (3)	Na—O^vi^	2.297 (3)
Te—O^i^	1.951 (3)	Na—O^vii^	2.297 (3)
Te—O^ii^	1.951 (3)	Na—O^viii^	2.297 (3)
Te—O^iii^	1.993 (3)	Na—O^iii^	2.545 (4)
Te—O^iv^	1.993 (3)	Na—O^iv^	2.545 (4)
Te—O^v^	1.993 (3)	Na—O^v^	2.545 (4)
			
O—Te—O^i^	95.42 (13)	O^vi^—Na—O^iii^	98.45 (11)
O—Te—O^ii^	95.42 (13)	O^vii^—Na—O^iii^	91.78 (15)
O^i^—Te—O^ii^	95.42 (13)	O^viii^—Na—O^iii^	156.33 (16)
O—Te—O^iii^	98.82 (19)	O^vi^—Na—O^iv^	91.78 (15)
O^i^—Te—O^iii^	79.08 (15)	O^vii^—Na—O^iv^	156.34 (16)
O^ii^—Te—O^iii^	165.14 (16)	O^viii^—Na—O^iv^	98.45 (11)
O—Te—O^iv^	79.08 (15)	O^iii^—Na—O^iv^	65.98 (14)
O^i^—Te—O^iv^	165.15 (16)	O^vi^—Na—O^v^	156.34 (16)
O^ii^—Te—O^iv^	98.82 (19)	O^vii^—Na—O^v^	98.45 (11)
O^iii^—Te—O^iv^	88.08 (14)	O^viii^—Na—O^v^	91.77 (15)
O—Te—O^v^	165.14 (16)	O^iii^—Na—O^v^	65.98 (14)
O^i^—Te—O^v^	98.82 (19)	O^iv^—Na—O^v^	65.98 (14)
O^ii^—Te—O^v^	79.08 (15)	Te—O—Te^iv^	100.92 (15)
O^iii^—Te—O^v^	88.08 (14)	Te—O—Na^ix^	120.25 (14)
O^iv^—Te—O^v^	88.08 (14)	Te^iv^—O—Na^ix^	123.88 (14)
O^vi^—Na—O^vii^	99.80 (15)	Te—O—Na^iv^	142.72 (16)
O^vi^—Na—O^viii^	99.80 (15)	Te^iv^—O—Na^iv^	87.65 (11)
O^vii^—Na—O^viii^	99.80 (15)	Na^ix^—O—Na^iv^	81.55 (11)

Symmetry codes: (i) −*x*+*y*, −*x*, *z*; (ii) −*y*, *x*−*y*, *z*; (iii) *x*−*y*−1/3, *x*−2/3, −*z*+1/3; (iv) −*x*+2/3, −*y*+1/3, −*z*+1/3; (v) *y*−1/3, −*x*+*y*+1/3, −*z*+1/3; (vi) −*x*+*y*+2/3, −*x*+1/3, *z*+1/3; (vii) −*y*−1/3, *x*−*y*−2/3, *z*+1/3; (viii) *x*−1/3, *y*+1/3, *z*+1/3; (ix) *x*+1/3, *y*−1/3, *z*−1/3.
